# Evaluation of Four Forensic Investigative Genetic Genealogy Analysis Approaches with Decreased Numbers of SNPs and Increased Genotyping Errors

**DOI:** 10.3390/genes15101329

**Published:** 2024-10-15

**Authors:** Yu Zang, Enlin Wu, Tingjun Li, Jiajun Liu, Riga Wu, Ran Li, Hongyu Sun

**Affiliations:** 1 Faculty of Forensic Medicine, Zhongshan School of Medicine, Sun Yat-Sen University, Guangzhou 510080, China; zangy3@mail2.sysu.edu.cn (Y.Z.); wuenlin3@mail2.sysu.edu.cn (E.W.); lity66@mail2.sysu.edu.cn (T.L.); liujj258@mail2.sysu.edu.cn (J.L.); wurg3@mail.sysu.edu.cn (R.W.); 2 Guangdong Province Translational Forensic Medicine Engineering Technology Research Center, Sun Yat-Sen University, Guangzhou 510080, China; 3 School of Medicine, Jiaying University, Meizhou 514015, China

**Keywords:** forensic investigative genetic genealogy (FIGG), single-nucleotide polymorphisms (SNP), kinship inference, identical by descent (IBD), method of moment (MoM)

## Abstract

**Background**: Forensic investigative genetic genealogy (FIGG) has developed rapidly in recent years and is considered a novel tool for crime investigation. However, crime scene samples are often of low quality and quantity and are challenging to analyze. Deciding which approach should be used for kinship inference in forensic practice remains a troubling problem for investigators. **Methods**: In this study, we selected four popular approaches—KING, IBS, TRUFFLE, and GERMLINE—comprising one method of moment (MoM) estimator and three identical by descent (IBD) segment-based tools and compared their performance at varying numbers of SNPs and levels of genotyping errors using both simulated and real family data. We also explored the possibility of making robust kinship inferences for samples with ultra-high genotyping errors by integrating MoM and the IBD segment-based methods. **Results**: The results showed that decreasing the number of SNPs had little effect on kinship inference when no fewer than 164 K SNPs were used for all four approaches. However, as the number decreased further, decreased efficiency was observed for the three IBD segment-based methods. Genotyping errors also had a significant effect on kinship inference, especially when they exceeded 1%. In contrast, MoM was much more robust to genotyping errors. Furthermore, the combination of the MoM and the IBD segment-based methods showed a higher overall accuracy, indicating its potential to improve the tolerance to genotyping errors. **Conclusions**: In conclusion, this study shows that different approaches have unique characteristics and should be selected for different scenarios. More importantly, the integration of the MoM and the IBD segment-based methods can improve the robustness of kinship inference and has great potential for applications in forensic practice.

## 1. Introduction

Two individuals who can be traced back (finite generations) to a common ancestor are related and are expected to share the same copies of the common ancestral DNA sequences, which are known as identical by descent (IBD) segments. In general, the more distant the relationship, the shorter the IBD segments they share. Hence, a relationship can be inferred from the IBD segments detected [1]. Based on this principle, forensic investigative genetic genealogy (FIGG), also known as long-range familial search, has emerged in recent years. It is considered a subdiscipline involving forensic genetics, genealogy, and bioinformatics [2,3,4] and differs greatly from the traditional kinship inference strategy in forensic genetics. Conventionally, a relationship is tested using dozens of short tandem repeats (STRs), which are obtained with capillary electrophoresis and then analyzed using likelihood ratio (LR) methods [4]. Due to the low power of common forensic STRs, relationships can only be reliably identified up to a second-degree relationship (e.g., uncle–nephew) [5]. In contrast, FIGG is based on dense single-nucleotide polymorphisms (SNPs), typically over 600,000, obtained from whole-genome sequencing or high-density microarrays, and can identify relatives as distant as those with a seventh-degree relationship (e.g., third cousins) [4,6].

The exploratory approaches are frequently used in FIGG and can be subdivided into the method of moment (MoM) and the IBD segment-based methods [4,6]. MoM estimates the coefficients of pairwise relatedness, such as the kinship coefficient (θ) and Cotterman coefficients, based on the observed identical by state (IBS) of the genetic markers [7,8], while the IBD segment-based methods infer relationships by identifying IBD segments between two individuals. Depending on whether alleles are assigned to paternal or maternal chromosomes, the IBD segment-based methods can be subdivided into two types: phased [9,10] and phase-free [11,12,13]. With phased genotyping data, if two fragments are identical at multiple continuous markers so that the probability of a random match is extremely low, the two fragments can be considered IBD segments [11,14], whereas with phased-free genotyping data, an IBD segment is assigned if two individuals share at least one half-genotype at multiple continuous markers and the length is longer than a certain threshold [12,13]. MoM is robust and computationally efficient, such as PLINK [7] and KING [8], while those based on the IBD segment-based methods are better for identifying distant relatives [6,15], such as IBIS [13] and GERMLINE [9].

However, in the face of complicated crime scene samples, deciding which approach to use remains a troubling problem for investigators. Previous studies have shown that some approaches performed unsatisfactorily for samples of suboptimal quality and quantity, where null alleles and genotyping errors occur frequently, and as a result, relationships were misclassified [16,17,18]. Therefore, selecting appropriate approaches can improve not only the efficiency but also the robustness of kinship inference. It is necessary to answer three questions before selection: (i) How many SNPs are required for each approach to provide effective information for kinship inference? (ii) What is the upper limit of an approach in terms of genotyping errors? More importantly, (iii) how can we improve the robustness using these existing approaches?

In this study, we selected four existing approaches and evaluated their performance in terms of decreased numbers of SNPs and increased genotyping error rates, aiming to help the selection of appropriate approaches in FIGG. The four selected approaches were KING [8], a common MoM estimator; IBIS [13], a popular phase-free IBD segment-based tool; TRUFFLE [11], a phase-free IBD segment-based tool, which embeds a model to deal with genotyping errors; and GERMLINE [9], a typical phased IBD segment-based tool. We first evaluated the performance of these tools using simulated datasets with decreasing numbers of SNPs from 5 million to 5000 and increasing genotyping error rates from 0.1% to 10%. Then, we explored the possibility of making robust kinship inferences for samples with ultra-high genotyping errors by integrating the MoM and IBD segment-based methods. Finally, we tested the performance on real diluted and degraded DNA samples.

## 2. Materials and Methods

### 2.1. Simulation

**Figure 1:**
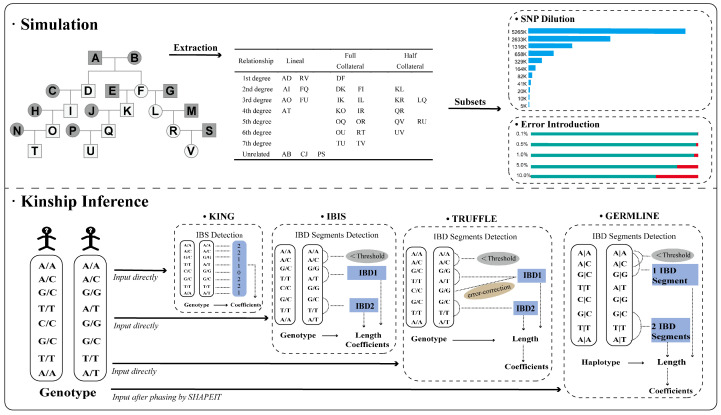
Workflow of pedigree simulation and kinship inference. In the part of simulation, individuals in grey represent the founders. In the part of error introduction, red bars represent the genotyping error level.

The haplotype data of 208 unrelated individuals were obtained from the 1000 Genomes Project (GRCh37) [19], encompassing 103 Han Chinese individuals in Beijing (CHB) and 105 Southern Han Chinese (CHS) individuals. PLINK [7] was then employed for SNP filtering. Specifically, only bi-allelic SNPs with a minor allele frequency (MAF) over 0.05 were included, and SNPs on the X chromosome, Y chromosome, and mitochondrial DNA were excluded. After filtering, 5,265,508 SNPs were retained, hereafter referred to as 5265 K. The 5265 K panel was then used as a reference dataset. Finally, pedigrees shown in Figure 1 were simulated by Ped-sim [20] using the sex-average genetic map created by Bhérer et al. [21] All the simulations were performed with default parameters unless otherwise specified. Since 11 founders (the individuals in gray shown in Figure 1) are required for each simulation, a maximum of 18 families could be created based on the 208 individuals. Therefore, we repeated the simulation 10 times by changing the seeds and finally obtained a total of 180 families, which consisted of lineal relatives as well as full and half collateral relatives. In each simulated family, 30 pairs were extracted (Figure 1) so that first- to seventh-degree relatives and unrelated pairs were included. In total, there were 540, 900, 1080, 720, 720, 540, and 360 pairs of first- to seventh-degree relatives, as well as 540 unrelated pairs. Throughout this study, inbred relationships were not considered.

Considering that the effective number of SNPs may change if we use different panels, different volumes of sequencing data, or samples of different quality and/or quantity, a series of subsets of the 5265 K panel were randomly selected, including 2633 K, 1316 K, 658 K, 329 K, 164 K, 82 K, 41 K, 20 K, 10 K, and 5 K. These subsets were used to determine a minimum panel, that is, a minimum number of SNPs that still had sufficient information for kinship inference. Then, based on the minimum panel, five more datasets with genotyping errors of 0.1%, 0.5%, 1%, 5%, and 10% were simulated using Ped-sim.

### 2.2. Mock Challenging Samples

With informed consent, the whole blood samples of six individuals from a Han Chinese family (Figure S1) were collected. The relationships had been confirmed using STR genotype data in our previous study [5]. With these family members, we had 4, 2, 1, 2, 3, 2, and 1 pairs of first- to seventh-degree relatives, respectively. DNA were then extracted using the QIAamp^®^ DNA Investigator Kit (Qiagen, Hilden, Germany) and quantified using the Qubit^®^ 3.0 fluorometer (Thermo Fisher Scientific, Waltham, MA, USA) with the Qubit dsDNA HS Assay Kit (Thermo Fisher Scientific, Waltham, MA, USA) according to the manufacturer’s protocol. To mock low-copy DNA samples, each DNA sample was diluted with ATE buffer, thus resulting in four samples with total DNA of 10 ng, 1 ng, 0.5 ng, and 0.1 ng. To mock degraded DNA samples, 100 ng of each DNA sample was fragmented using a Covaris M220 Focused-ultrasonicator^™^ following the manufacturer’s recommendation. By applying different parameters, we had four degraded DNA samples with average fragment sizes of 1500 bp, 800 bp, 400 bp, and 150 bp, respectively. Finally, six intact, 24 diluted, and 24 degraded DNA samples were genotyped using the Infinium Asian Screening Array (ASA, Illumina, San Diego, CA, USA). This panel consists of ~650 K SNPs specifically selected from East Asian populations. Genotyping data were then refined using VCFtools [22], i.e., bi-allelic SNPs with an MAF above 0.05 and Hardy–Weinberg equilibrium *p*-values above 0.000001 were used as inclusion criteria.

### 2.3. Kinship Inference

Four representative approaches for kinship inference––one MoM estimator, KING [8], and three IBD segment-based software tools, IBIS [13], TRUFFLE [11], and GERMLINE [9]—were employed. To facilitate the comparison, the kinship coefficient (θ) was used to determine the degree of relatedness. We expanded the empirical criteria described before [8] to seventh-degree relationships and defined more distant relatives as unrelated pairs (Table 1). Kinship coefficients were estimated using the four tools as follows:

(1) KING estimates the proportion of IBD segments based on the IBS status of a large number of genetic markers. Since there is no need to specify allele frequency, genotyping data are directly input, and KING directly outputs the kinship coefficient, hereafter referred to as $\theta_{KING}$.

(2) As a phase-free IBD segment detection tool, IBIS identifies IBD segments based on IBS status. The minimum length and number of markers required for defining a segment as IBD were set as 2 cM and 186, respectively. In addition, genetic positions were interpolated before input based on the sex-average genetic map [21]. Although IBIS was used for IBD detection, it also outputs kinship coefficients directly (using the flag-printCoef), hereinafter referred to as $\theta_{IBIS}$.

(3) Similar to IBIS, TRUFFLE also estimates IBD segments based on IBS status. There are two types of IBD segments, IBD1 and IBD2. The IBD1 segment refers to haploid matches between any pair of individuals where only a pair of haplotypes are involved, while IBD2 segments are diploid matches where both haplotypes of a pair of individuals match [23]. Two criteria are required for defining a segment as IBD: (i) a probability of match at continuous markers that is too low to be caused by random similarity and (ii) a length of a segment that is long enough to be less likely caused by linkage disequilibrium. Here, the two thresholds were set as 10^−8^ for both IBD1 and IBD2 and as 5 Mb for IBD1 and 2 Mb for IBD2. Importantly, a built-in error model is implemented in TRUFFLE, which can correct potential genotyping errors. TRUFFLE outputs both IBD1 and IBD2 segments, and with the two types of IBD segments, we could easily calculate three Cotterman coefficients (κ0, κ1, κ2) as:

$$\kappa 1=\frac{L\left(\mathrm{IBD}1\right)}{L\left(\mathrm{genome}\right)}$$

$$\kappa 2=\frac{L\left(\mathrm{IBD}2\right)}{L\left(\mathrm{genome}\right)}$$

κ0=1−κ1−κ2,

where L(IBD1) and L(IBD2) are the lengths of IBD1 and IBD2 segments summed across all autosomes and L(genome) is the total genetic length (approximately 3346.30 cM, according to the genetic map we adopted in this study). Then, the kinship coefficient was calculated as:

$$\theta =\frac{\kappa 2}{2}+\frac{\kappa 1}{4},$$

hereafter referred to as $\theta_{TRUFFLE}$.

(4) Since the IBD detection of GERMLINE is based on haplotype, genotype data have to be phased before inputting. In this study, phasing was performed using SHAPEIT v2 [24] with the sex-average genetic map [21]. The minimum length for identifying a segment as IBD was set to 3 cM, and ultimately, GERMLINE outputs all IBD segment information. Then, the Cotterman coefficients (κ0, κ1, κ2) and the kinship coefficient could be calculated as described above, hereafter referred to as $\theta_{GERMLINE}$.

To evaluate and compare the performance of different approaches, four indicators were calculated: overlapping rate, sensitivity (Sen), positive predictive value (PPV), and accuracy (AC). The overlapping rate is the proportion of pairs whose estimated kinship coefficients are within the reference range intervals estimated based on the reference panel. Sen was defined as the proportion of known relationships that are correctly inferred, and PPV was defined as the proportion of inferred relationships that are correctly assigned. Accuracy (AC) was defined as the proportion of all known relationships that are correctly inferred. All of the following analyses, including figure generation, were performed in R software v.4.0.3.

## 3. Results

### 3.1. Performance Using Panels with Different Numbers of SNPs

**Figure 2:**
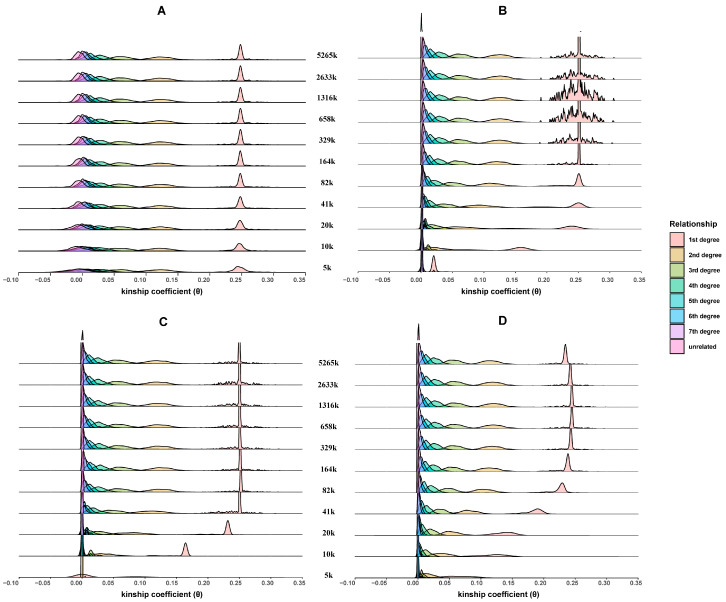
Distributions of kinship coefficients (θ) of first- to seventh-degree and unrelated relationships for different numbers of SNPs using KING (**A**), IBIS (**B**), TRUFFLE (**C**), and GERMLINE (**D**).

Figure 2 shows the distribution of estimated θ values for different panels and approaches. As expected, kinship coefficients for all the relationships fluctuated around the theoretical values (Table 1), but the four approaches performed differently. Specifically, $\theta_{KING}$ showed little change compared with that of the 5265 K panel, even at the smallest number of SNPs (5 K), regardless of increased variations. In contrast, although $\theta_{IBIS}$, $\theta_{TRUFFLE}$, and $\theta_{GERMLINE}$ remained largely unchanged with above 164 K SNPs, they showed larger variations and gradually shifted to the left with fewer SNPs. As a result, the kinship coefficients estimated by IBIS, TRUFFLE, and GERMLINE overlapped and became difficult to distinguish for different relationships. Notably, the value of $\theta_{KING}$ may be less than 0, while there is a lower limit (0) for the values of $\theta_{IBIS}$, $\theta_{TRUFFLE}$, and $\theta_{GERMLINE}$ since they are converted from the length of IBD segments.

**Figure 3:**
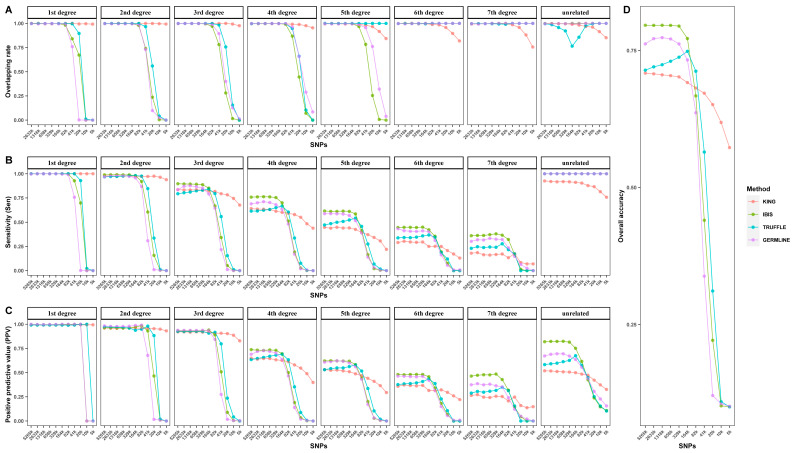
Performance of subsets with different numbers of SNPs using four approaches: (**A**) overlapping rate; (**B**) sensitivity (Sen); (**C**) positive predictive value (PPV); (**D**) overall accuracy (AC).

To quantitively evaluate the performance of the four approaches with these virtual panels (ranging from 5265 K to 5 K), the overlapping rate, Sen, PPV, and AC were assessed, and the results are shown in Figure 3. These four indicators showed a downward trend as the number of SNPs decreased but differed for different approaches. For KING, it had low Sen and PPV for fourth-degree or more distant relationships when large numbers of SNPs were used, thus resulting in low overall accuracy. However, the four indicators all decreased slowly and gradually with decreasing SNPs, indicating that KING is robust in decreasing SNP numbers. In contrast, IBIS, TRUFFLE, and GERMLINE all showed a sharp turning point, except for a few interesting changes (explained below). Specifically, the turning points of both Sen and PPV were observed at 82 K for first- and second-degree relationships and at 164 K for third- to seventh-degree relationships, suggesting that 164 K may be a minimum number for effective kinship inference. With respect to overlapping rates, when IBIS and GERMLINE were used, there were also turning points at 164 K for first- to fifth-degree relationships, but the overlapping rates remained unchanged for sixth- and seventh-degree relationships, as well as for unrelated pairs. The latter can be explained by the small θ values and large overlapping areas for these relationships. For the three types of relationships, the lower bounds were all 0, and they had similar upper bounds regardless of the number of SNPs (Figure S2), thus resulting in overlapping rates all close to 1 (Figure 3). Similar results were observed for first- to seventh-degree relationships when TRUFFLE was employed. However, an unexpected sharp decrease and then an increase were observed at 164 K for unrelated pairs. Indeed, for each relationship, $\theta_{TRUFFLE}$ was slightly underestimated compared to the theoretical values when using the 5265 K panel, and when using the 164 K panel, $\theta_{TRUFFLE}$ was distributed as expected (Figure S2). We speculated that when the number of SNPs was redundant, the underlying linkage disequilibrium may cause θ to be overestimated. This is why TRUFFLE performed best with the 164 K panel rather than with the 5265 K panel (Figure 3D). In fact, the authors of TRUFFLE may have considered this problem and recommend pruning markers to 100–500 K before inputting. “https://adimitromanolakis.github.io/truffle-website/index.html (accessed on 10 November 2023)”.

On the whole, when the number of SNPs was sufficient (more than 164 K), IBIS performed best, with an AC equal to 0.796–0.772, followed by GERMLINE (AC = 0.774–0.733), TRUFFLE (AC = 0.749–0.714), and KING (AC = 0.709–0.691). Therefore, IBIS shows superiority over the other three methods, especially for the inference of distant relationships. When the number of SNPs was approximately 82 K, all the approaches performed comparably, with TRUFFLE reaching the best AC (0.712). However, when the number of SNPs was even smaller, only KING (AC = 0.672–0.573) made relatively effective inferences.

In summary, decreasing the number of SNPs has little effect on kinship inference for both the MoM and IBD segment-based methods when the number of SNPs is more than 164 K, at which point overlapping rates for all relationships are all over 0.99 for each approach (except for unrelated pairs using TRUFFLE). The effect becomes non-negligible for IBD segment-based methods when the number of SNPs is below 164 K. Therefore, we considered the 164 K subset as the minimum panel for kinship inference and employed it for genotyping error analyses.

### 3.2. Performance Using Panels at Different Levels of Genotyping Error

**Figure 4:**
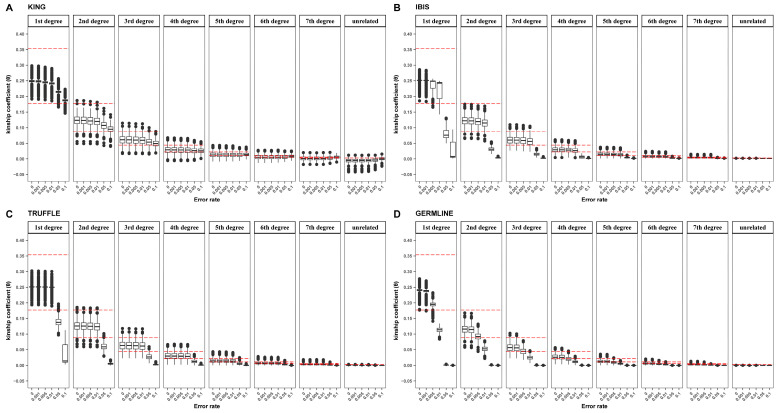
Kinship coefficients (θ) of first- to seventh-degree and unrelated relationships based on the 164 K panel with different genotyping errors using KING (**A**), IBIS (**B**), TRUFFLE (**C**), and GERMLINE (**D**). Red dashed line shows empirical criteria (predefined in Table 1) for kinship inference.

As shown in Figure 4, the estimated kinship coefficients changed accordingly with increasing genotyping errors, and a high genotyping error rate caused a significant reduction in θ values. We found that $\theta_{KING}$, $\theta_{IBIS}$, $\theta_{TRUFFLE}$, and $\theta_{GERMLINE}$ remained essentially unchanged at low error rates (0.1%), whereas the four tools performed differently when genotyping errors increased. Specifically, $\theta_{KING}$ values of first- to fourth-degree relationships tended to decrease slightly, while those of fifth-degree or more distant relationships (including unrelated pairs) tended to increase. Interestingly, reduced variations were observed when the error rate exceeded 1%. In contrast, $\theta_{IBIS}$, $\theta_{TRUFFLE}$, and $\theta_{GERMLINE}$ all tended to decrease when the error rates exceeded 0.1%, 1%, and 0.1%, respectively. Furthermore, the higher the genotyping error rate, the bigger the difference in kinship coefficients for the four approaches. By leveraging this phenomenon, we attempted to correct the reduction in kinship coefficients caused by genotyping errors. First, we averaged the θ values estimated by the four approaches when the genotyping error rate was 0 and took it (named “expected kinship coefficient”) as the dependent variable. Then, 13 variables were constructed, including $\theta_{\mathrm{KING}}$, $\theta_{\mathrm{IBIS}}$, $\theta_{\mathrm{TRUFFLE}}$, $\theta_{\mathrm{GERMLINE}}$, $\theta_{\Delta \left(\mathrm{KING},\mathrm{IBIS}\right)}$, $\theta_{\Delta \left(\mathrm{KING},\mathrm{TRUFFLE}\right)}$, $\theta_{\Delta \left(\mathrm{KING},\mathrm{GERMLINE}\right)}$, $\theta_{\Delta \left(\mathrm{IBIS},\mathrm{TRUFFLE}\right)}$, $\theta_{\Delta \left(\mathrm{IBIS},\mathrm{GERMLINE}\right)}$, $\theta_{\Delta \left(\mathrm{TRUFFLE},\mathrm{GERMLINE}\right)}$, $\theta_{\left(\mathrm{KING}/\mathrm{IBIS}\right)}$, $\theta_{\left(\mathrm{TRUFFLE}/\mathrm{IBIS}\right)}$, and $\theta_{\left(\mathrm{GERMLINE}/\mathrm{IBIS}\right)}$, and stepwise multiple linear regression was performed with a 10-fold cross-validation using the packages *caret*, *leaps*, and *MASS* in R. Aside from the four parameters $\theta_{\mathrm{KING}}$, $\theta_{\mathrm{IBIS}}$, $\theta_{\mathrm{TRUFFLE}}$, $\theta_{\mathrm{GERMLINE}}$, other variables were constructed in order to measure the difference in output values between these approaches:

$$\theta_{\Delta \left(A,B\right)}=\theta_{A}-\theta_{B}$$

$$\theta_{\left(A/B\right)}=\theta_{A}÷\theta_{B},$$

where A and B each represent one of the four approaches. Note that since IBIS calculates θ with a supplemental kinship coefficient factor (default = 0.00138), only $\theta_{\mathrm{IBIS}}$ will not be 0, and is therefore considered the denominator when calculating $\theta_{\left(A/B\right)}$. As shown in Table 2, several models were constructed, and we found that the model M7 fit the best, hereafter referred to as the combination.

**Figure 5:**
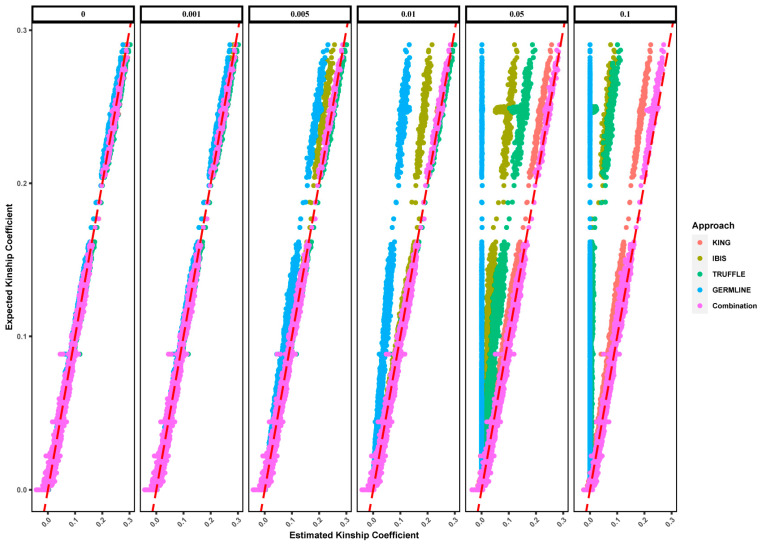
Estimated kinship coefficients through four respective approaches and combinations. Red dashed line shows the square of the correlation coefficient (r^2^) between expected and estimated kinship coefficients is 1.

This model was quite robust regarding genotyping errors, and the estimated kinship coefficients were maintained throughout the error ranges (Figure S3). As shown in Figure 5, the $\theta_{\mathrm{combination}}$ values were closer to the expected values than those of any single approach alone, especially when the genotyping error rate exceeded 1%. Given this, integrating MoM and IBD segment-based methods has the potential to improve the tolerance to genotyping errors.

**Figure 6:**
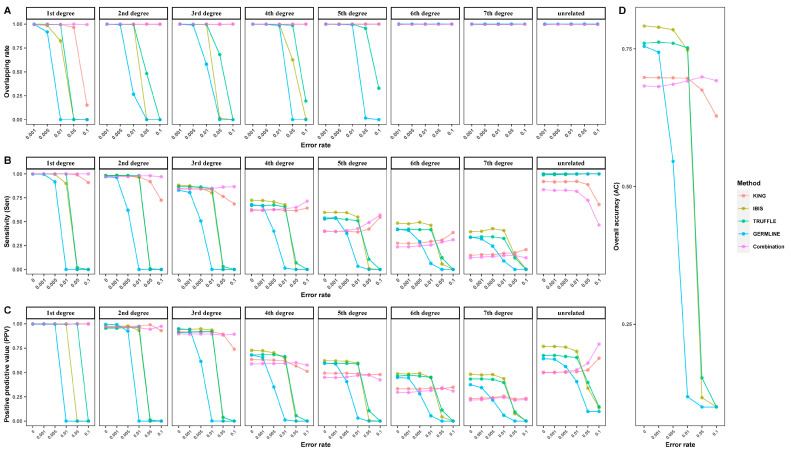
Performance of subsets with different genotyping error rates using four approaches and combinations: (**A**) overlapping rate; (**B**) sensitivity (Sen); (**C**) positive predictive value (PPV); (**D**) overall accuracy (AC).

Again, in order to quantitively evaluate performance in the presence of genotyping error for the four tools and our newly established model, the overlapping rate (treating 164 K SNP panel with no genotyping errors as the reference panel), Sen, PPV, and AC were assessed. As shown in Figure 6, these four indicators all remained stable when the error rate was low. When the genotyping error rate increased further, these four indicators of KING and the combination method remained stable, while those of IBIS, TRUFFLE, and GERMLINE showed a sharp decrease at the 0.05 error rate, indicating that KING and the combination method are more robust to genotyping error. Notably, Sen decreased gradually with increasing genotyping error rates for first- to third-degree as well as unrelated relationships but increased for fourth- to seventh-degree relationships. This was consistent with the distributions of $\theta_{KING}$ in Figure 4, which showed narrow variations and were slightly overestimated with high genotyping error. A similar explanation can also be applied for the trends of overlapping rates, which remained close to 1 as the genotyping error rate increased, except for first-degree pairs with KING. In addition, the overlapping rates for IBIS, TRUFFLE, and GERMLINE also remained close to 1 for distant relationships. This could be explained by a failure of IBD segment detection and a large proportion of θ equal to the lower limit (0).

In terms of accuracy, when the genotyping error rate was relatively low, IBIS outperformed the other three tools. When the error rates increased to 1%, TRUFFLE performed better and had a higher AC. However, when the error rates increased to 5%, even the tolerant KING showed a marked decrease in AC. Interestingly, our newly established model, the combination method, showed very stable ACs under different levels of genotyping error, which suggests that the model is reliable for identifying close relationships when the genotyping error rate is ultra-high.

Overall, increasing the rate of genotyping errors has significant effects on kinship inference, especially when the rate exceeds 1%. MoM and the IBD segment-based method performed differently in response to genotyping errors. KING was the best performer, followed by TRUFFLE, IBIS, and GERMLINE. In addition, by taking advantage of the difference in the estimated kinship coefficients of the four tools, we showed that integrating MoM and the IBD segment-based methods can improve the tolerance to genotyping errors.

### 3.3. Performance Using Real Samples

**Figure 7:**
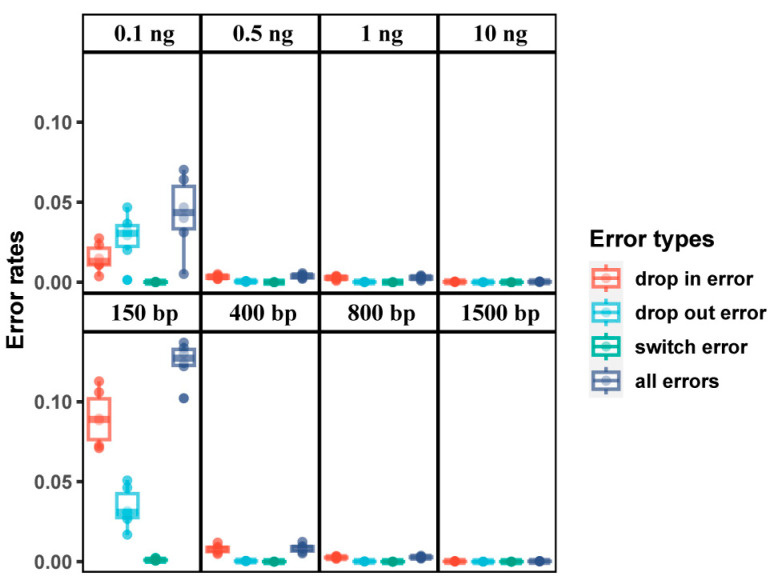
The rates and types of genotyping errors for the diluted and degraded DNA samples.

Ultimately, six intact (100 ng), 24 diluted (10 ng, 1 ng, 0.5 ng, and 0.1 ng), and 24 degraded (1500 bp, 800 bp, 400 bp, and 150 bp) DNA samples were employed to assess the performance of each approach on challenging samples. After genotyping and filtering, 302,756 SNPs were retained. Subsequently, the original DNA as a reference and the rates and types of genotyping errors for these diluted and degraded DNA samples were estimated (Figure 7). Three types of errors were studied: (1) drop in error, which occurs when an additional allele is reported, e.g., from “AA” to “AG”; (2) drop out error, which occurs when an allele is absent from the sample, e.g., from “AG” to “AA”; and (3) switch error, which occurs when opposite homozygous genotypes are reported, e.g., from “AA” to “GG”. As shown in Figure 7, an increase in error rate was observed with the reduction of DNA input and with the increasing levels of degradation. When the amount of input DNA exceeds 0.5 ng or the length of the DNAs fragment surpasses 400 bp, the error rates were relatively low (<0.012). The predominant type of genotyping error in these instances was drop in error. However, a notable increase in error rates (up to 0.070) was observed when the amount of input DNA decreased to 0.1 ng, and the dominant error type changed to drop out. Furthermore, a significant escalation in error rates is also observed when the length of the DNA fragments decreased to 150 bp, with an overall error rate as high as 0.137.

**Figure 8:**
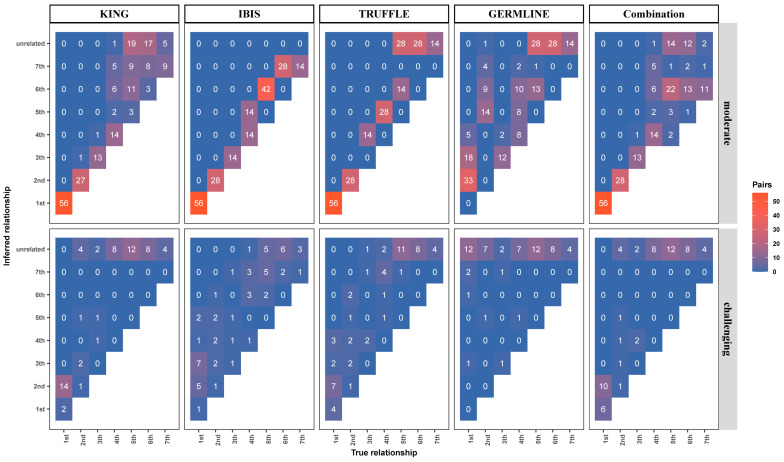
Kinship inference of diluted and degraded samples using the four approaches studied and the combination method.

Generally, we can obtain high-quality DNA samples from a reference (either a reference sample from a database or the person of interest), but crime scene samples are mostly of low quality. Therefore, we paired each intact DNA with corresponding diluted or degraded samples, resulting in 72, 36, 18, 36, 54, 36, and 18 pairs of first- to seventh-degree relationships, respectively. The kinship coefficient estimation and kinship inference of these pairs were performed using KING, IBIS, TRUFFLE, GERMLINE, and the combination method, respectively (Figure S4). Figure 8 illustrates that under moderate quality or quantity conditions (input DNA ≥ 0.5 ng or average fragment length ≥ 400 bp), KING, IBIS, TRUFFLE, and the combination method performed comparably and correctly identified most close relationships (up to second-degree relationships). However, GERMLINE performed rather poorly, possibly due to phasing errors caused by high missing data and forced phasing (using the flag -force through SHAPEIT). For distant relationships (fifth- to seventh-degree relationships), all approaches incorrectly identified most pairs. TRUFFLE and GERMLINE misidentified most pairs (70 pairs) as unrelated, while KING and IBIS correctly identified 15 and 14 pairs. The combination method achieved the highest number of correct identifications (17 pairs) but misidentified 38 pairs as relationships within one degree of difference and 28 pairs as unrelated. Under more challenging conditions, i.e., when input DNA decreased to 0.1 ng or the average fragment length decreased to 150 bp, correct identifications were limited to a few close relationships. The combination method performed best for first-degree relationships, correctly identifying six out of sixteen pairs, followed by TRUFFLE with four, KING with two, IBIS with one, and GERMLINE with none. For second-degree relationships, all approaches except GERMLINE correctly identified only one out of eight pairs.

In summary, when dealing with samples of moderate quality or quantity (genotyping error rates below 1%), KING and IBIS have better performance and correctly identify most of the first- to fourth-degree relationships. However, when faced with more challenging samples (genotyping error rates close to 5–10%), the combination method outperformed the existing tools for close kinship inference.

## 4. Discussion

MoM and the IBD segment-based method are both widely used for kinship inference by scientists and investigators. However, there is still a lack of evaluation regarding which approach should be used in forensic practice. In this study, we compared the performance of four common tools given different numbers of SNPs and different levels of genotyping error and explored the potential to improve the tolerance to genotyping errors by integrating MoM and the IBD segment-based method.

All four approaches had high stability when the number of SNPs was larger than 164 K, and three IBD segment-based tools had higher accuracy in identifying distant relationships, which was consistent with previous studies [15,25]. However, with fewer than 82 K SNPs, only KING could provide relatively reliable inferences, albeit with significantly reduced efficiency compared to scenarios with more SNPs. It should be noted that although the efficiency of all four approaches decreased as the number of SNPs decreased, the reasons are slightly different. KING, as a method of moment (MoM) estimator, estimates the proportion of IBD segments based on the number of identity-by-state SNP markers. As the number of SNPs decreases, the random effect increases, and the estimated kinship coefficients show greater variation, causing KING to show a gradual decrease in efficiency. In contrast, IBIS, TRUFFLE, and GERMLINE, as IBD segment-based tools, identify segments based on specific thresholds related to IBD length, the distance between markers, and/or the number of markers. As the number of SNPs decreases, more IBD segments fail to meet the thresholds. In addition, as the number of markers decreases, the distance between SNPs also increases and may be greater than the threshold, which may cause an IBD segment to be interrupted and fail to pass the IBD length threshold. All these factors can lead to a decrease in the efficiency of IBD detection and subsequent kinship inference.

Generally, if a sample is of good quality, the output data are expected to have low genotyping error. For these samples, if fewer than 82 K SNPs are obtained, MoM estimators (e.g., KING) are recommended, whereas if more than 164 K SNPs are obtained, IBD segment-based tools (e.g., IBIS) are preferred. In contrast, if a sample is of poor quality and is expected to have a high genotyping error, both MoM and the IBD segment-based methods exhibit a significant decline, especially when the genotyping error rate exceeds 1%. However, MoM is more appropriate than the IBD segment-based methods for these challenging samples. We also showed that the combination of MoM and the IBD segment-based methods could improve the accuracy of identifying close relationships, even at ultra-high genotyping error rates (above 5%). Therefore, if the sample quality is uncertain, we recommend adopting both MoM estimators and IBD segment-based tools. More importantly, if there is a big difference in the estimated kinship coefficients between the two types of methods, we recommend adopting models that combine all the outputs of the related tools.

Several notes need to be made. First, although GERMLINE is considered one of the most accurate methods [25], its use in forensic practice is questionable. As demonstrated in Section 3.3, when dealing with genotype data of high missing rates, phasing may introduce additional errors, which in turn may decrease the accuracy. Given that the quality of forensic samples is often unknown and varies significantly, GERMLINE may not be suitable for forensic DNA analysis. In addition, although both simulated data and mock challenging samples showed that the combined method had an increased accuracy for challenging samples, the model was based on simulated datasets of 164 K SNPs and with genotyping error rates of 0–10%. However, in forensic practice, the genotyping error rates may exceed this boundary and have reduced performance. Finally, although adjusting parameters may theoretically lead to better performance, previous studies showed that there was no combination of reasonably permissive parameters that could rescue the performance of existing methods when the genotyping error was in the 1–5% range [15].

Furthermore, the analyses of real samples indicated that it was still difficult to correctly infer relationships with 0.1 ng DNA or with DNA of average fragment lengths of 150 bp. Therefore, there is an urgent need to develop new algorithms to address this issue. For example, Snedecor et al. [26] proposed an IBD-based method that was accurate up to fifth-degree relatives using only 10,000 SNPs. This windowed kinship algorithm uses thresholds that are slightly lower than theoretical values, making it also relatively robust to genotyping error. Additionally, there are also several tools, such as optimized MOM algorithms and machine learning [27], that have been developed and have shown their robustness in recent years. These methods are promising and will be investigated in our future work. In addition, methods designed for ancient DNA may also be promising alternative tools in FIGG [28].

Benefitting from the development of public databases, FIGG has grown rapidly since the well-known Golden State killer case was solved and has been used to solve hundreds of active and cold cases [29]. However, there are still many problems to be addressed. For example, different algorithms are used for different databases and the performance needs to be evaluated, especially with respect to crime scene samples. In addition, since investigators need to upload high-density SNP data of case samples to large-scale public databases [30,31,32], concerns about privacy and data loss have been raised [33,34,35]. Therefore, maintaining a balance between privacy protection and efficient application is also an issue that needs to be addressed in the future [36,37].

## 5. Conclusions

The existing methods are sufficient for kinship inference (first- to seventh-degree and unrelated relationships) using genotyping data with more than 164 K SNPs and less than 1% genotyping error. MoM estimators need significantly fewer SNPs and are more robust for genotyping errors, while IBD segment-based methods are more effective in identifying distant relationships. If a sample is of good quality, IBD segment-based tools such as IBIS are preferred; otherwise, MoM estimators should be used. In addition, the combination of both types of methods could improve performance when the genotyping error is high, and it is promising for challenging forensic samples. This study sheds light on how to select the appropriate method based on the number of SNPs or the genotyping error rate in FIGG or a complex.

## Figures and Tables

**Table 1:** Empirical criteria of kinship inference based on kinship coefficient (θ).

Relationships	Expected θ	Threshold
Twins/same individual	$\frac{1}{2}$	$\geq \frac{1}{2^{3/2}}$
First-degree	$\frac{1}{4}$	$\left[\frac{1}{2^{5/2}},\frac{1}{2^{3/2}}\right)$
Second-degree	$\frac{1}{8}$	$\left[\frac{1}{2^{7/2}},\frac{1}{2^{5/2}}\right)$
Third-degree	$\frac{1}{16}$	$\left[\frac{1}{2^{9/2}},\frac{1}{2^{7/2}}\right)$
Fourth-degree	$\frac{1}{32}$	$\left[\frac{1}{2^{11/2}},\frac{1}{2^{9/2}}\right)$
Fifth-degree	$\frac{1}{64}$	$\left[\frac{1}{2^{13/2}},\frac{1}{2^{11/2}}\right)$
Sixth-degree	$\frac{1}{128}$	$\left[\frac{1}{2^{15/2}},\frac{1}{2^{13/2}}\right)$
Seventh-degree	$\frac{1}{256}$	$\left[\frac{1}{2^{17/2}},\frac{1}{2^{15/2}}\right)$
Unrelated	0	$<\frac{1}{2^{17/2}}$

**Table 2:** Models for θ estimation by combining different tools.

Tools	Model	$\theta_{\mathrm{combination}}$	R^2^
KING and IBIS	M1	$0.00248+0.98133\times \theta_{\mathrm{IBIS}}$ $+1.24292\times \theta_{\Delta \left(KING,IBIS\right)}-0.00010\times \theta_{\left(\mathrm{KING}/\mathrm{IBIS}\right)}$	0.99024
KING and TRUFFLE	M2	$0.00209+0.99867\times \theta_{\mathrm{TRUFFLE}}$ $+1.27598\times \theta_{\Delta \left(\mathrm{KING},\mathrm{TRUFFLE}\right)}$	0.99064
KING and GERMLINE	M3	$0.00210+1.15566\times \theta_{\mathrm{KING}}$ $+0.20366\times \theta_{\mathrm{GERMLINE}}$	0.98890
KING and IBIS and TRUFFLE	M4	$0.00279+0.99651\times \theta_{\mathrm{IBIS}}+1.35786\times \theta_{\Delta \left(\mathrm{KING},\mathrm{IBIS}\right)}\;\;\;\;+0.37875\times \theta_{\Delta \left(\mathrm{IBIS},\mathrm{TRUFFLE}\right)}-0.00036\times \theta_{\left(KING/IBIS\right)}\;\;\;\;-0.00002\times \theta_{\left(\mathrm{TRUFFLE}/\mathrm{IBIS}\right)}$	0.99146
KING and IBIS and GERMLINE	M5	$0.00308+0.98074\times \theta_{\mathrm{IBIS}}+1.22371\times \theta_{\Delta \left(\mathrm{KING},\mathrm{GERMLINE}\right)}\;\;\;\;-1.18864\times \theta_{\Delta \left(\mathrm{IBIS},\mathrm{GERMLINE}\right)}-0.00007\times \theta_{\left(\mathrm{KING}/\mathrm{IBIS}\right)}\;\;\;\;-0.00195\times \theta_{\left(\mathrm{GERMLINE}/\mathrm{IBIS}\right)}$	0.99040
KING and TRUFFLE and GERMLINE	M6	$0.00202+1.25589\times \theta_{\mathrm{KING}}$ $-0.20160\times \theta_{\mathrm{TRUFFLE}}-0.07663\times \theta_{\mathrm{GERMLINE}}$	0.99115
KING and IBIS and TRUFFLE and GERMLINE	M7	$0.00279+0.98496\times \theta_{\mathrm{TRUFFLE}}+1.24569\times \theta_{\Delta \left(\mathrm{KING},\mathrm{IBIS}\right)}\;\;\;\;+0.09770\times \theta_{\Delta \left(\mathrm{KING},\mathrm{GERMLINE}\right)}\;\;\;\;+1.43790\times \theta_{\Delta \left(\mathrm{IBIS},\mathrm{TRUFFLE}\right)}-0.00034\times \theta_{\left(\mathrm{KING}/\mathrm{IBIS}\right)}\;\;\;\;+0.00021\times \theta_{\left(\mathrm{TRUFFLE}/\mathrm{IBIS}\right)}\;\;\;\;-0.00114\times \theta_{\left(\mathrm{GERMLINE}/\mathrm{IBIS}\right)}$	0.99194

## Data Availability

The data presented in this study are available upon request from the corresponding author. The data are not publicly available due to the restriction of privacy and law.

## Supplementary Materials

The following supporting information can be downloaded at: https://www.mdpi.com/article/10.3390/genes15101329/s1, Figure S1: The pedigree of six samples; Figure S2: Kinship coefficients (θ) of first- to seventh-degree and unrelated relationships for different numbers of SNPs using KING (A), IBIS (B), TRUFFLE (C), and GERMLINE (D). Red dashed line shows empirical criteria (predefined in Table 1) for kinship inference; Figure S3: Distributions of kinship coefficient (θ) of first- to seventh-degree and unrelated relationships based on subsets of 164 K SNPs with increasing genotyping errors using combination of four approaches. Red dashed line shows empirical criteria (predefined in Table 1) for kinship inference; Figure S4: Estimated kinship coefficients and inferred results of real samples using four approaches and the combination method: (A) KING; (B) IBIS; (C) TRUFFLE; (D) GERMLINE; (E) Combination. In each subplot, the scatter plots show the distribution of estimated kinship coefficients for sample pairs using nine conditions (five different total DNA amounts and four different target average fragment lengths), and the green dots represent correct inference while the red dots represent incorrect inference.
