# Supplementary material for: Evaluation of Four Forensic Investigative Genetic Genealogy Analysis Approaches with Decreased Numbers of SNPs and Increased Genotyping Errors
Source: Genes (Basel). 2024 Oct 15;15(10):1329. doi: 10.3390/genes15101329 (PMC11507463; doi:10.3390/genes15101329)
Supplement: Supplementary file 1 [file genes-15-01329-s001.zip › genes-3195334-supplementary.pdf]

Evaluation of four forensic investigative genetic genealogy analysis approaches with decreased numbers of SNPs and increased genotyping errors

Supplementary materials

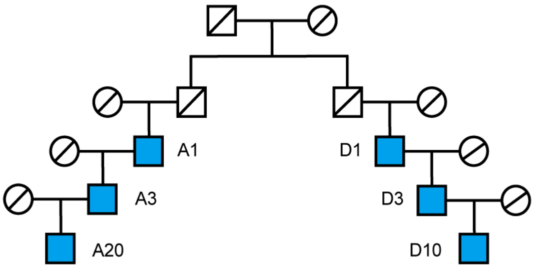

Figure S1. The pedigree of six samples

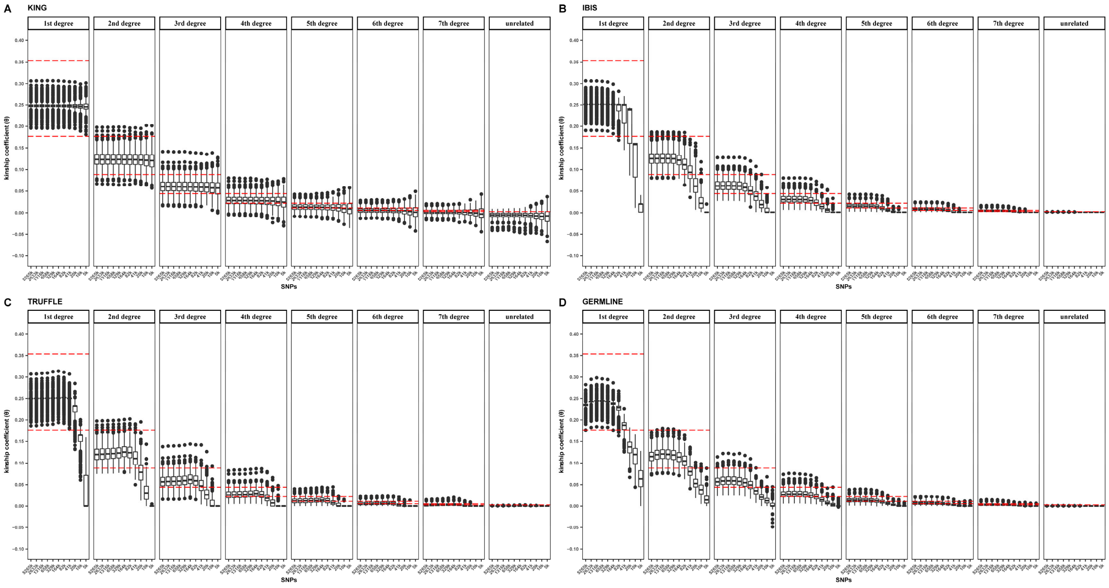

Figure S2. Kinship coefficients (θ) of first- to seventh-degree and unrelated relationships for different numbers of SNPs using KING (A), IBIS (B), TRUFFLE (C), and GERMLINE (D). Red dashed line shows empirical criteria (predefined in Table 1) for kinship inference.

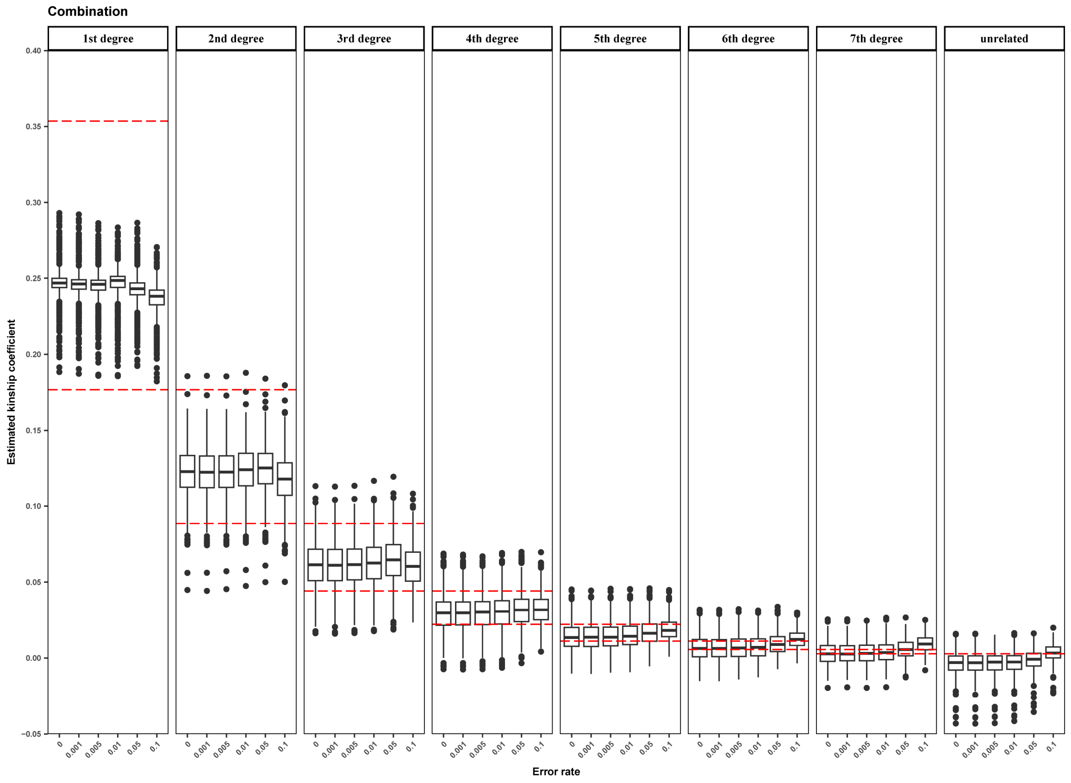

Figure S3. Distributions of kinship coefficient (θ) of first- to seventh-degree and unrelated relationships based on subsets of 164K SNPs with increasing genotyping errors using combination of four approaches. Red dashed line shows empirical criteria (predefined in Table 1) for kinship inference.

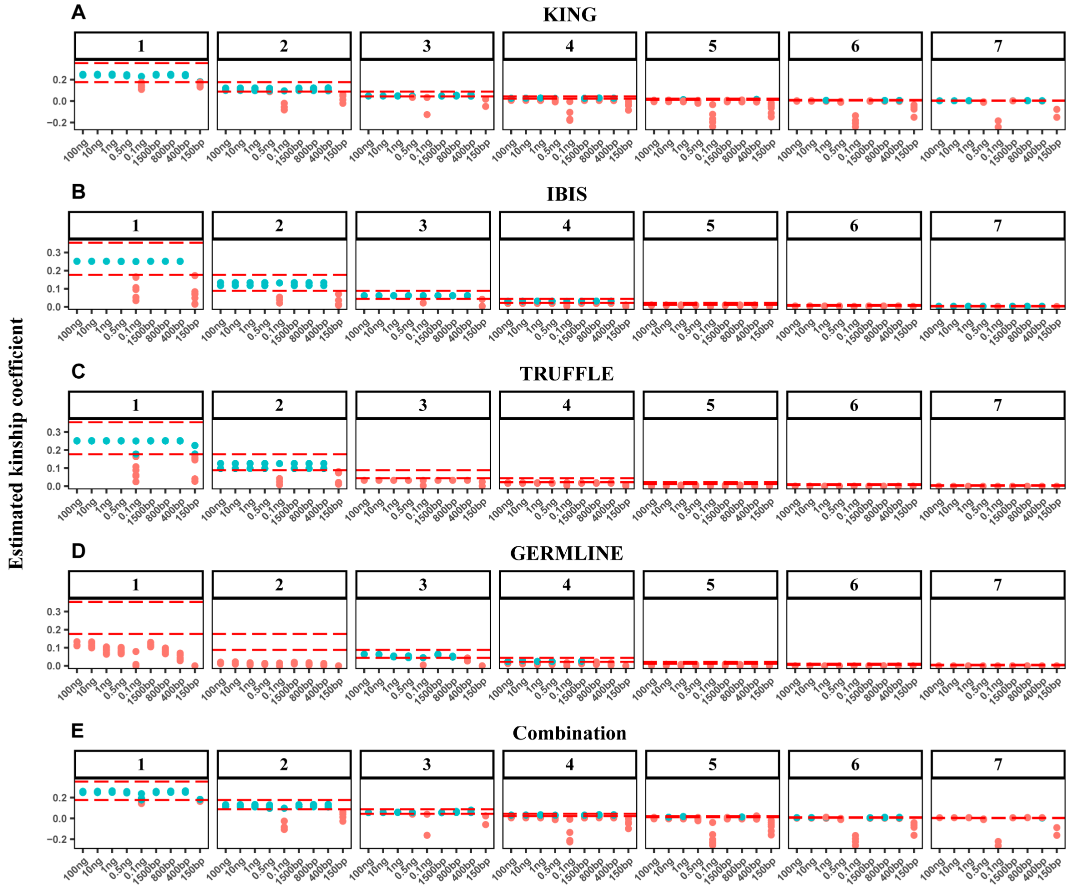

Figure S4. Estimated kinship coefficients and inferred results of real samples using four approaches and the combination method: (A) KING; (B) IBIS; (C) TRUFFLE; (D) GERMLINE; (E) Combination. In each subplot, the scatter plots show the distribution of estimated kinship coefficients for sample pairs using nine conditions (five different total DNA amounts and four different target average fragment lengths), and the green dots represent correct inference while the red dots represent incorrect inference.
